# The impact of psychological attachment on the relationship between periodontal health and dental fear in patients with versus without psoriasis: a questionnaire-based, cross-sectional study

**DOI:** 10.1186/s12903-021-01457-8

**Published:** 2021-03-04

**Authors:** Christian Graetz, Sirka Woeste, Ullrich Mrowietz, Johannes C. Ehrenthal

**Affiliations:** 1 Clinic of Conservative Dentistry and Periodontology, University of Kiel, Arnold-Heller-Str. 3, Haus B, 24105 Kiel, Germany; 2 Psoriasis-Center at the Department of Dermatology, University Medical Center Schleswig-Holstein, Campus, Kiel, Germany; 3 Department of Psychology, University of Cologne, Cologne, Germany

**Keywords:** Psoriasis, Periodontal health, Dental fear, Psychological attachment, Questionnaire

## Abstract

**Background:**

While there is increasing evidence for the relevance of psychosocial variables such as dental fear or psychological attachment in dentistry, much less is known about the mechanisms that determine the strength of those associations. One potential moderator is the occurrence of a comorbid chronic disease such as psoriasis, which is linked to relevant disease parameters such as periodontal inflammation. The aim of the study was to test a moderation model of the relationship between dental fear, psychological attachment and psoriasis on periodontal health.

**Methods:**

A total of 201 patients (100 with psoriasis, 101 without psoriasis) were included in a questionnaire-based, cross-sectional study. Dental status was measured with the Community Periodontal Index (CPI), dental fear was measured with the Hierarchical Anxiety Questionnaire (HAQ), and psychological attachment was measured with the Relationship Questionnaire (RQ). In addition to the examination of main effects, bootstrapping-based analyses were conducted to test the moderating influence of psychological attachment on the association between CPI and dental fear, gain moderated by group (with vs. without psoriasis).

**Results:**

Controlling for several covariates, higher CPI scores were associated with higher levels of dental fear only in individuals without psoriasis under conditions of higher levels of psychological attachment anxiety and lower levels of attachment avoidance.

**Conclusion:**

In individuals without psoriasis, psychological attachment can moderate the association between periodontal health and dental fear. This may provide a useful framework for reducing dental fear through interventions on the level of the dentist-patient relationship.

## Background

Periodontal disease is a common medical condition [1] that is characterized by a chronic bacterial infection of the tissue surrounding the teeth, which, if untreated, leads to a loss of the diseased tooth through the irreversible destruction of the periodontium [2]. In particular, its chronic forms need to be understood from a biopsychosocial perspective [3]. Psychosocial risk factors for chronic dental diseases include a direct and indirect impact of psychiatric disorders such as depression [4, 5], treatment-specific parameters such as dental fear [6], and long-standing psychosocial styles that influence coping with diseases, patient-dentist interaction, and stress regulation, as described, for example, by psychological attachment theory [7].

Dental fear is linked to subjective as well as objective measures of periodontal health and wellbeing. Patients with high dental fear may expect, experience, and try to avoid oral interventions or self-care due to anticipatory avoidance of pain [8, 9]. This is especially relevant for patients with a problematic periodontal status: if individuals with a high risk of periodontal disease also experience high levels of dental fear, it can prevent them from initiating or continuing necessary treatment. At the same time, individuals differ in how they deal with fear and other negative emotions. A model that is increasingly often used in medicine to understand these interindividual differences is provided by psychological attachment theory.

Drawing on concepts and findings from evolutionary and developmental psychology and advancing classical psychoanalytic motivational psychology, attachment theory describes how individuals cope with insecurity and perceived threat across the lifespan (for an overview from a medical perspective, see Hunter and Maunder [10]). Depending on repeated interactions with primary caregivers, different attachment styles start to develop in children and form relatively stable patterns of perception, cognition, emotion, and regulation with regard to the self and others across the life span. These patterns can either represent what is called attachment security, a social-cognitive configuration characterized by a fundamental trust that others will be there in times of need, combined with relatively adequate self-regulation, or by attachment insecurity. For the latter, there are two associated basic regulatory styles. Attachment anxiety describes constant hyperactivation of attachment-related cognitions and wishes at the price of dependency and hyperarousal, while attachment avoidance describes a constant downplaying of those factors and needs at the price of staying alone [11]. In medicine, attachment styles influence illness perception, coping mechanisms, compliance, stress regulation, and pain perception [12–16]. In patients with periodontal disease, attachment insecurity (i.e., higher levels of anxiety or avoidance) was associated with potentially adverse health behavior, readiness for treatment, and treatment utilization [7].

At the same time, the impact of attachment anxiety and avoidance on the regulation of emotions such as fear and stress sometimes depends on other factors that influence the functioning of the attachment system. For example, individuals with high attachment avoidance are usually very proficient at suppressing attachment-related cognitions and seem less stressed than individuals with high levels of attachment anxiety. However, when avoidant individuals are exposed to cognitive or attachment-related emotional stressors, these seemingly positive effects disappear [17, 18]. In other words, while there is evidence that psychological attachment influences how individuals process information or cope with illness, including periodontal disease, it remains unclear under what conditions this effect is especially relevant. Cognitive load may affect attachment-related performance in experimental tasks as described above, and longstanding psychological factors interact with attachment regarding stress physiology [12]. Less is known about the influence of a stressor such as a co-occurring somatic disease on the relationship between psychological attachment and health-related variables. For periodontal health, an especially suitable condition is psoriasis [19].

Psoriasis is a chronic inflammatory disease of the skin with high prevalence rates worldwide [20]. Psoriasis and periodontal diseases share common risk factors such as smoking [21, 22], obesity [23–25], diabetes, and psychosocial variables [5, 22, 26–30]. Even if both diseases differ in severity and progression at the level of the individual patient, there are corresponding genetic predispositions for the regulation of the immune system [31, 32]. However, there is also evidence that psoriasis is not directly associated with general attachment insecurity or other psychological variables [33]. This qualifies psoriasis as a stressor for research on conditions that determine the influence of psychological attachment on factors related to periodontal health.

The aim of our study was to test the moderation effect of psychological attachment on the link between periodontal status and dental fear in adults with and without psoriasis. We assessed dental fear and attachment styles in groups with varying levels of periodontal health and psoriasis. Our hypothesis was that psoriasis may modify the role of psychological attachment on the association between periodontal status and dental fear. Due to the novelty of the research question, we had no a priori prediction of the direction of the results.

## Methods

For the current study, 201 patients with (n = 100) and without psoriasis (n = 101) were investigated in a mixed clinical and questionnaire-based survey.^1^ Participants were consecutively included in the study after approval of the study protocol by the local institutional review board (Kiel: D570/15) and had to fulfill all inclusion criteria as well have as sufficient knowledge of the German language to answer a set of questionnaires on psychosocial and health-related variables. Participants were recruited at two dermatological doctor offices and the Department of Dermatology, University of Kiel. Further recruitment details are available in a previously published study that investigated the clinical parameters of psoriasis and periodontitis [34].

### Demographic and health related data

The severity level of psoriasis was recorded and measured by the psoriasis area and severity index (PASI), and periodontal health was measured by the community periodontal index (CPI) [35]. CPI was determined for each sextant and classified as follows: score 0, 1, and 2: "no periodontitis"; score 3 and 4: "periodontitis possible". We used the highest score of each participant as a quantitative index for impaired periodontal heath. To control for general oral health, the DMFT index assessed decayed, missing, and filled teeth for each participant (wisdom teeth were not included). Other variables relevant for the current study as covariates were sex (0 = female, 1 = male), age (years at time of the study), levels of oral hygiene (self-reported brushing of teeth, 0 = not every day, 1 = once a day, 2 = twice a day or more), smoking (0 = never smokers, 1 = former smokers, 3 = current smokers), and utilization style (0 = no preventative dental visits, 1 = preventative dental visits).

### Psychosocial variables: attachment theory and dental fear

We assessed attachment style with the dimensional German version of the Relationship Questionnaire (RQ), which correlates substantially with other instruments that capture adult attachment style [36]. The RQ measures current self-concept with regard to attachment by ratings of four short vignettes, which each represents a quadrant of the model by Bartholomew and Horowitz [37]; i.e., secure, dismissing, preoccupied, and fearful. Each vignette is rated on a seven-point scale from one (disagree strongly) to seven (agree strongly). The RQ is widely used in attachment research and represents an easy-to-administer instrument that does not depend on attachment expectations from romantic relationships [38]. In line with current recommendations [39, 40], we do not categorize patients on the basis of their questionnaire values into categorical attachment styles but rather calculated an attachment anxiety dimension by subtracting the secure plus dismissing scales from the fearful plus preoccupied scales and an attachment avoidance dimension by subtracting the secure plus preoccupied scales from the fearful plus dismissing scales.

Dental fear was assessed with the Hierarchical Anxiety Questionnaire (HAQ; [41]). The HAQ is an eleven-item questionnaire that asks for a rating on a scale from one (relaxed) to five (sick with fear) in relation to statements describing a prototypical dentist visit from thinking about the visit (item one “How do you feel when you imagine you have to go to the dentist tomorrow?) to an intervention (item 11 “One of your wisdom teeth is to be removed; the injection has already been given. The dentist picks up the scalpel.”). The HAQ is widely used in studies and everyday practice in German-speaking countries and has shown good psychometric properties and validity [42]. All questionnaires were given blind to dental status. To further ensure data integrity, they were returned to, prepared for analysis, and merged with the dental data at the nontreating site.

### Statistical analyses

**Fig. 1:**
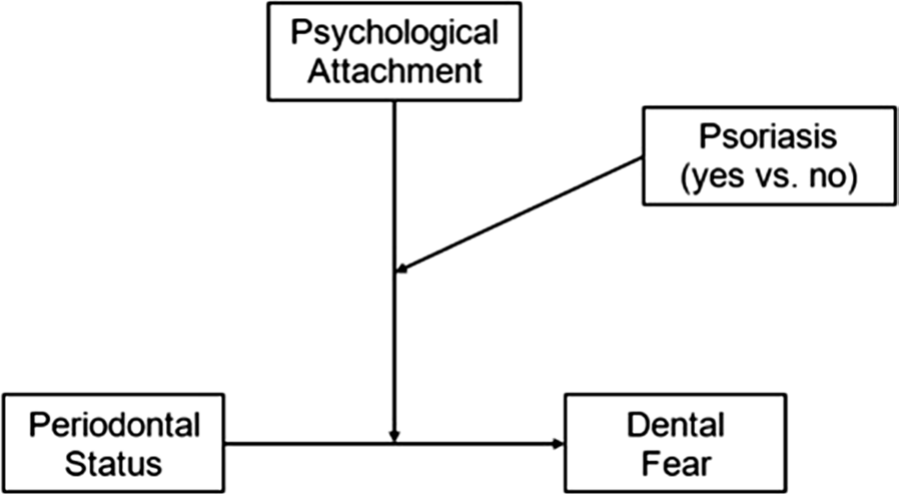
Conceptual diagram of the study

To test our hypothesis of different psychological attachment-related dynamics on the association between periodontal health and dental fear depending on the presence or absence of psoriasis, we conducted bootstrapping-based (number of bootstrap samples = 1000) moderated moderation analyses (for a conceptual diagram, see Fig. 1), which is especially robust for the given data structure.^2^ We simultaneously controlled for some a priori chosen covariates, such as age at baseline, smoking status, sex, oral status, levels of oral hygiene, and dentist utilization style, to address the potential impact of current risk factors. All analyses were conducted with IBM SPSS 23 and the PROCESS macro version 3 [43].

## Results

### Overall sample and psoriasis

The sample consisted of 99 women and 102 men, with a mean age of 47.1 ± 15.7 years; 30.35% were identified as smokers, 32.34% as former smokers, and 37.31% as never having smoked. Of the participants, 4.97% (n = 10) reported suffering from diabetes.

In the psoriasis group, 68% of the participants had severe psoriasis according to the PASI, and 32% had a less severe form. Sixty-six percent received systemic therapy, 25% received topical therapy, and 9% currently received no therapy.

### Oral health and dental visits

In the overall sample, 80.6% of the participants reported regular utilization of dental visits/professional care, and 18.9% only went to the dentist when experiencing complaints. A total of 75.12% brushed their teeth ≥ 2 times a day, 23.38% brushed their teeth once a day, and two participants reported not brushing every day. Nearly half (49.25%) of all 201 participants cleaned interdental areas regularly.

Just 2.5% of all participants reported having received periodontal treatment prior to the study. Thirty-nine participants reported bleeding of their gum after brushing, 159 reported no bleeding, and three participants gave no information.

According to the CPI, 20.4% of all participants showed code 4 in ≥ 1 sextant, code 3 in 30.4%, code 2 in 22.9%, and code 1 in 25.4%. More information on the sample can be found in Table 1.

**Table 1:** Univariate group differences

Variable	Group with psoriasis	Group without psoriasis	χ^2^
	N	N	
Sex			
Male	59	43	5.42*
Female	41	58	
Dental care utilization style			
When experiencing discomfort	21	17	0.52
Regular control appointments	79	83	
	M (SD)	M (SD)	F
HAQ dental fear	21.23 (10.36)	16.37 (9.13)	1.81
CPI	2.92 (.98)	2.01 (1.00)	42.12***
RQ attachment anxiety	− 3.08 (3.13)	− 4.24 (3.76)	5.36*
RQ attachment avoidance	− 1.29 (3.33)	− 1.39 (2.30)	0.05
Age	47.38 (14.68)	46.90 (16.78)	0.05
Smoking	.81 (.78)	1.33 (.79)	21.96***
Dental hygiene	1.65 (.52)	1.84 (.37)	9.40**
DMFT score	13.67 (6.87)	11.66 (7.16)	4.11*

*HAQ* hierarchical anxiety questionnaire, *CPI* community periodontal index, *RQ* relationship questionnaire, *DMFT* decayed, missing, filled teeth index, *N* number of

***p > 0.001, **p > 0.01, *p > 0.05, ‘*p* > 0.10

### Group differences

In exploratory univariate ANOVAs and χ2 tests, patients with comorbid psoriasis had significantly higher CPI, higher DMFT scores, higher levels of psychological attachment anxiety, and lower scores concerning smoking but also worse dental hygiene habits and a comparably higher male-to-female ratio. There were no significant differences in dental care utilization style, dental fear, age, or psychological attachment avoidance (see Table 1).

### Moderation analysis

Regarding our main hypothesis, there was a differential impact of psychological attachment on the association between periodontal status and dental fear, depending on whether individuals suffered from comorbid psoriasis.

**Fig. 2:**
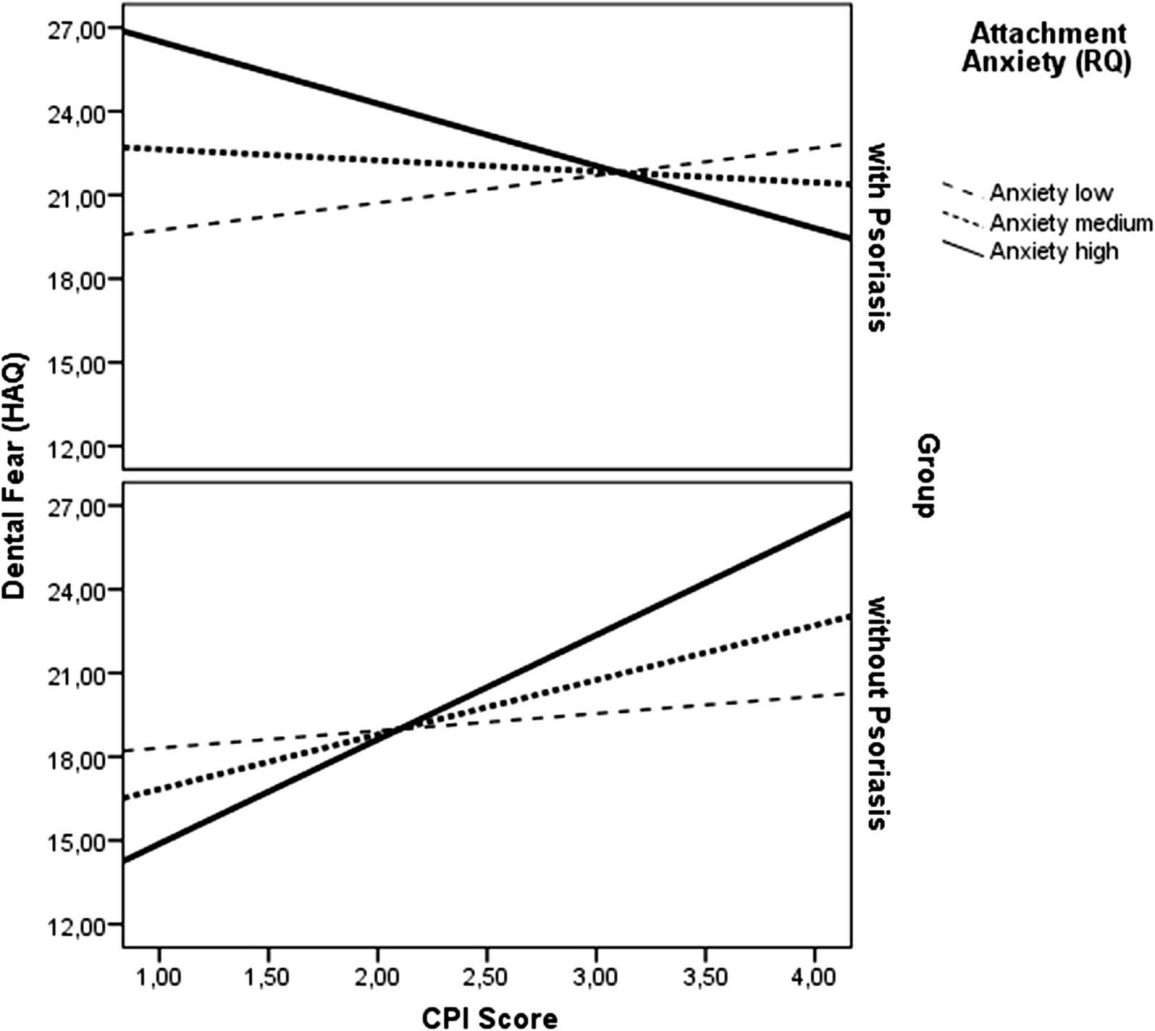
Moderation of the association between periodontal status according to the CPI score and dental fear by psychological attachment anxiety in individuals with versus without psoriasis. *Note*: *HAQ* hierarchical anxiety questionnaire, *CPI* community periodontal index, *RQ* relationship questionnaire

The model testing the influence of attachment anxiety showed a significant main effect for group, a significant two-way interaction for CPI × group, and a significant three-way-interaction effect for CPI × group × attachment anxiety. None of the other main or interaction effects were statistically significant, controlling for a number of covariates (see Table 2, Fig. 2). In other words, with increasing levels of attachment anxiety, there was a larger association between CPI and dental fear, especially in the group without psoriasis compared to the group with psoriasis (see Table 2, Fig. 2).

**Table 2:** Moderation of the association between periodontal health (CPI) and dental fear (HAQ) by psychological attachment anxiety in individuals with versus without psoriasis

Variable	Coefficient	*t*	LLCI	ULCI
CPI	− 2.23	− 1.39	− 5.39	0.93
RQ attachment anxiety	1.43	1.33	− 0.69	3.55
Group (psoriasis yes/no)	− 17.60	− 2.90**	− 29.60	− 5.60
CPI × RQ attachment anxiety	− 0.46	− 1.26	− 1.18	0.26
CPI × group	5.97	2.59*	1.43	10.52
RQ attachment anxiety × group	− 2.36	− 1.88’	− 4.84	0.12
CPI × group × RQ attachment anxiety	0.91	1.98*	0.003	1.81
*Covariates*				
RQ attachment avoidance	− 0.48	− 2.17*	− 0.91	− 0.04
Age	− 0.21	− 3.29**	− 0.34	− 0.08
Smoking	1.71	1.91’	− 0.05	3.47
Sex	− 5.78	− 4.05***	− 8.59	− 2.97
Levels of oral hygiene	− 0.32	− 0.20	− 0.52	2.89
Utilization style	− 0.86	− 2.12*	− 7.47	− 0.26
DMFT score	0.27	1.87’	− 0.02	0.55

*HAQ* hierarchical anxiety questionnaire, *CPI* community periodontal index, *RQ* relationship questionnaire, *DMFT* decayed, missing, filled teeth index, *LLCI* lower level of confidence interval, *ULCI* upper level of confidence interval

***p < 0.001; **p < 0.01; *p < 0.05; ‘p < 0.1

**Fig. 3:**
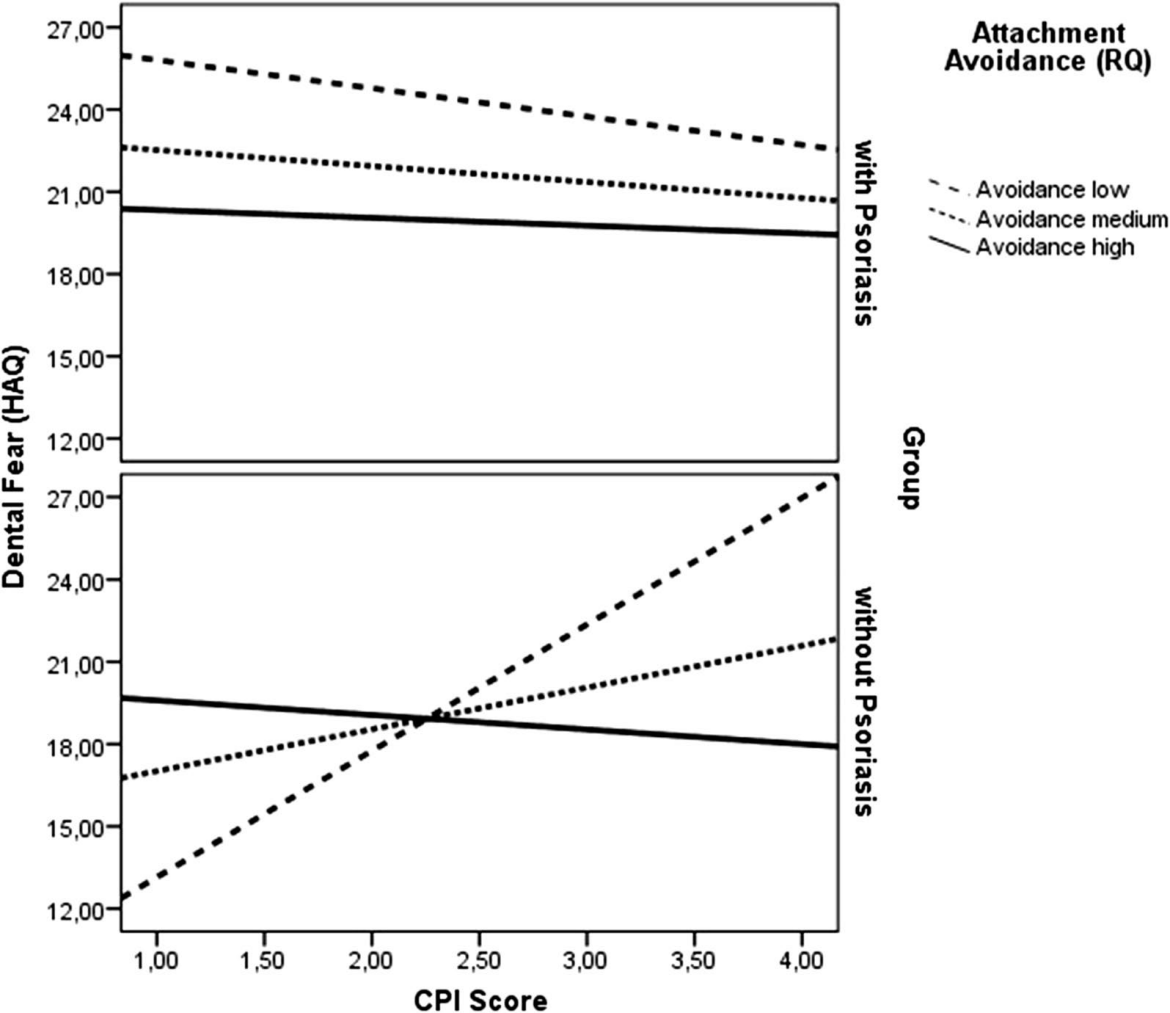
Moderation of the association between periodontal status according to the CPI score and dental fear by psychological attachment avoidance in individuals with versus without psoriasis. *Note: HAQ* hierarchical anxiety questionnaire, *CPI* community periodontal index, *RQ* relationship questionnaire

The model testing the influence of attachment avoidance showed a significant two-way interaction of RQ attachment avoidance by group and a significant three-way interaction of CPI by group by RQ attachment avoidance. None of the other main or interaction effects were statistically significant, controlling for a number of covariates (see Table 1). In other words, under conditions of relatively lower levels of attachment avoidance, higher CPI scores were associated with higher levels of dental fear, especially in the group without psoriasis compared to the group with psoriasis (see Table 3, Fig. 3).

**Table 3:** Moderation of the association between periodontal health (CPI) and dental fear (HAQ) by psychological attachment avoidance in individuals with versus without psoriasis

Variable	Coefficient	t	LLCI	ULCI
CPI	− 0.43	− 0.39	− 2.61	1.74
RQ attachment avoidance	− 1.24	− 1.19	− 3.31	0.82
Group (psoriasis yes/no)	− 4.06	− 1.01	− 12.01	3.90
CPI × RQ attachment avoidance	0.15	0.45	− 0.50	0.79
CPI × group	0.93	0.63	− 2.00	3.87
RQ attachment avoidance × group	3.56	2.65**	− 0.91	6.21
CPI × group × RQ attachment avoidance	− 1.18	− 2.46*	− 2.12	− 0.23
*Covariates*				
RQ attachment anxiety	0.21	1.06	− 0.18	0.59
Age	− 0.21	− 3.34**	− 0.34	− 0.09
Smoking	1.99	2.28*	0.27	3.72
Sex	− 5.25	− 3.85***	− 7.94	− 2.55
Levels of oral hygiene	− 0.02	− 0.01	− 3.14	3.10
Utilization style	− 4.56	− 2.52*	− 8.12	− 0.99
DMFT score	0.25	1.78’	− 0.03	0.53

*HAQ* hierarchical anxiety questionnaire, *CPI* community periodontal index, *RQ* relationship questionnaire, *DMFT* decayed, missing, filled teeth index, *LLCI* lower level of confidence interval, *ULCI* upper level of confidence interval

***p < 0.001; **p < 0.01; *p < 0.05, ‘*p* < 0.1

## Discussion

To the best of our knowledge, this is the first attempt to test attachment-related dynamics in dentistry in a sample of individuals with versus without a comorbid somatic condition such as psoriasis. We found psychological attachment insecurity as measured by more avoidance or anxiety to moderate the association between the severity of periodontal symptoms and dental fear, especially in individuals without psoriasis. Under conditions of relatively higher attachment anxiety but lower attachment avoidance, higher levels of CPI were associated with higher levels of dental fear. This points to the conclusion that a comorbid chronic medical condition may alter the regulatory influence of psychological attachment on factors associated with periodontal health.

From a psychological perspective, this is relevant, as it provides insight into mechanisms that determine when and how patients experience higher levels of treatment-specific fear in dentistry. Higher attachment anxiety relates to a habitual hyperactivation of relationship-oriented wishes, a lower threshold regarding social exclusion and abandonment, and a neglect of personal competence [10]. The attachment system is also associated with self-esteem and health behavior [44], which may be additionally influenced by periodontal status. Therefore, the findings on the impact of high attachment anxiety (and low attachment avoidance) in the group without psoriasis represent what would be expected by the model.

As this is the first study to test these associations in a sample of individuals with psoriasis, the psychological dynamics in this group are less clear. What we can tentatively suggest in line with our results is that the expected impact of attachment anxiety on the association between periodontal health and dental fear disappears. This could be related to some baseline differences regarding risk factors. While we controlled for each of those statistically, they could still influence attachment- or disease-related dynamics. It is also important to note that although the individuals with psoriasis reported less smoking than the nonpsoriasis group, they brushed their teeth less regularly, although it would be advisable to do so. In addition, individuals with psoriasis had higher levels of psychological attachment anxiety. Higher levels of attachment anxiety in the context of psoriasis could be related to changes in self-image due to coping with a chronic disease [45], fear of stigmatization or rejection due to the disease [46], and perhaps effects of parental worrying on developmental factors for individuals with psoriasis [47].

Other possibilities why the effect of attachment on the association between periodontal health and dental fear disappears relate to specific health-behavior and the doctor–patient relationship. It can be assumed that for individuals with psoriasis, there is a general effect of being more used to medical appointments, which does not necessarily need to be shown in lower levels of dental fear but rather in knowing that avoiding contact with physicians is not a viable strategy. One could also speculate that the person of the physician can have a different meaning for patients with a chronic disease such as psoriasis. Especially for individuals with higher attachment anxiety, psoriasis patients may identify dentists as quasi-attachment figures that provide security rather than elicit fear. The psychological mechanisms in patients with psoriasis need to be better understood to develop interventions that help to reduce dental fear. For the individuals in the nonpsoriasis group, for example, dental fear may be reduced by a tailored dentist-patient relationship that acknowledges the patients’ need for closeness in individuals with high attachment anxiety and could also help to reduce unnecessary appointments [7]. This is needed in clinical practice, as treating progressive diseases such as periodontitis and psoriasis will lead to more complex therapies [48, 49]. Therefore, it is necessary to remove barriers and improve collaborations between dentists and other areas of medicine, including mental health and other psychological factors [50].

### Limitations

There are a number of limitations to our study. First, participants were recruited from different settings. While recruiting was performed carefully and strictly according to predefined inclusion and exclusion criteria, a statistically significant difference in some variables between both groups remained [34]. Second, although this was the first study on this topic, we did not assess the levels of dental and psychosocial parameters at the beginning of psoriasis treatment. Despite the cited evidence on the development of attachment tendencies or dental fear due to disease factors (e.g., developing other phobias), we cannot rule out potential influences of similar or other elements in our study. The third limitation relates to sample size. While 201 patients seem a fair number for a nonfunded study, it limits the detection of small effects of possible predictor variables and the use of advanced statistical modeling techniques such as testing the moderated moderation hypothesis at the level of latent instead of observed variables. The initial power calculation for the study was performed to answer a different research question [34]. However, bootstrapping-based approaches are usually quite robust, even for sample sizes smaller than ours [51]. In addition, the naturalistic nature of the data, including somatic multimorbidity, does not permit a detailed analysis of which somatic diseases may have the most deleterious influence on the patients’ mood. We also do not have information about adverse life events or other factors that could have influenced mental health (e.g., depression) over the longitudinal course of psoriatic and/or dental treatment or detailed assessment of socioeconomic status [52]. Our associations are based on cross-sectional data. Future studies could make use of either momentary ecological assessment or experimental manipulation of a stimulus (i.e., situational provoking dental fear) to better capture related dynamics over time. We also cannot rule out that our findings are due to other mechanisms not assessed in our study. For example, we do not know if the current tooth level is a result of treatment or the fear of interventions. Another possible factor is the influence of shame, which tends to increase patients’ failure to disclose medically relevant information to clinicians [53]. In addition, the results of our moderation model should not be generalized for other categories of patients with comorbid diseases without more research.

## Conclusion

Our results stress the importance of further psychosomatic research in dermatology and dentistry. For most patients without psoriasis, dentists should pay attention to psychological attachment needs, especially concerning attachment anxiety and related hyperactivation, as they may be connected to higher dental fear. Future studies should investigate whether a specific, attachment-oriented intervention can reduce dental fear in these patient populations. Additionally, further research is needed on attachment-related dynamics among individuals with psoriasis. Therefore, we recommend a closer collaboration between dermatology, dentistry, and psychosomatic medicine for better understanding and ultimately treating both diseases.

## Data Availability

The datasets generated and analyzed during the current study are not publicly available due the guidelines of the data protection regulation of the university hospital Schleswig–Holstein, Campus Kiel but are available from the corresponding author on reasonable request.

## Abbreviations

CPI: Community periodontal index
DMFT: Decayed, missing, filled, teeth index
HAQ: Hierarchical anxiety questionnaire
LLCI: Lower level of confidence interval
PASI: Psoriasis area and severity index
RQ: Relationship questionnaire
SPSS: Statistical package for social sciences
ULCI: Upper level of confidence interval

## Footnotes

1 For the current analyses, due to some individual missing data in the questionnaire files, the sample size varied between 201 and 189.

2 In detail, the analyses conducted under the umbrella of ‘conditional process analysis’ make use of OLS regression, testing the related effects simultaneously, while applying bootstrapping, i.e., a resampling procedure that allows for a better estimation of the sampling distribution and therefore related confidence intervals.
